# The Impact of Coronary Artery Disease on Outcomes in Patients With Peripartum Cardiomyopathy

**DOI:** 10.7759/cureus.59269

**Published:** 2024-04-29

**Authors:** Omar Elkattawy, Jay V Phansalkar, Sherif Elkattawy, Omar Mohamed, Jahanzeb Javed, Afif Hossain, Kulsum Larry, Shriya Patel, Yash Shah, Fayez Shamoon

**Affiliations:** 1 Internal Medicine, Rutgers University New Jersey Medical School, Newark, USA; 2 Cardiothoracic Surgery, Rutgers University New Jersey Medical School, Newark, USA; 3 Cardiology, St. Joseph's University Medical Center, Paterson, USA; 4 Medicine, Saint Barnabas Medical Center, Livingston, USA; 5 Radiology, Rutgers University New Jersey Medical School, Newark, USA

**Keywords:** coronary artery disease, peripartum cardiomyopathy, clinical cardiology, cardiovascular prevention, cardio-obstetrics, outcomes of peripartum cardiomyopathy, cad: coronary artery disease

## Abstract

Introduction

The purpose of this study was to determine the prevalence of coronary artery disease (CAD) among patients admitted with peripartum cardiomyopathy (PPCM) as well as to analyze the independent association of CAD with in-hospital outcomes among PPCM patients.

Methods

Data were obtained from the National Inpatient Sample from January 2016 to December 2019. We assessed the independent association of CAD with outcomes in patients admitted with PPCM. Predictors of mortality in patients admitted with PPCM were also analyzed.

Results

There was a total of 4,730 patients with PPCM, 146 of whom had CAD (3.1%). Multivariate analysis demonstrated that CAD in patients with PPCM was independently associated with several outcomes, and, among them, ST-segment elevation myocardial infarction (STEMI) (adjusted odds ratio (aOR): 58.457, 95% CI: 5.403-632.504, p= 0.001) was positively associated with CAD. CAD was found to be protective against preeclampsia (aOR: 0.351, 95% CI: 0.126-0.979, p = 0.045). Predictors of in-hospital mortality for patients with PPCM include cardiogenic shock (aOR: 12.818, 95% CI: 7.332-22.411, p = 0.001), non-ST elevation myocardial infarction (NSTEMI) (OR: 3.429, 95% CI: 1.43-8.22, p = 0.006), chronic kidney disease (OR: 2.851, 95% CI: 1.495-5.435, p = 0.001), and atrial fibrillation (OR: 2.326, 95% CI: 1.145-4.723, p = 0.020).

Conclusion

In a large cohort of patients admitted with PPCM, we found the prevalence of CAD to be 3.1%. CAD was associated with several adverse outcomes, including STEMI, but protective against preeclampsia.

## Introduction

Peripartum cardiomyopathy (PPCM) is an uncommon form of heart disease that affects mothers near the end of pregnancy or in the months following delivery. It is a poorly understood entity, and, in general, is considered a diagnosis of exclusion. The Heart Failure Association of the European Society of Cardiology defines PPCM as heart failure with an ejection fraction < 45% secondary to left ventricular systolic dysfunction toward the end of pregnancy or in the months following delivery, where no other cause of heart failure is found [1]. While PPCM remains an idiopathic disease, it is known that greater age, black race, preeclampsia, and hypertension are associated with an increased risk of developing the condition [2]. Nutritional deficiencies, viral myocarditis, and autoimmune processes have been proposed as possible etiologies; however, evidence for these is not robust [3]. Studies using mouse models have provided evidence for a vascular-hormonal etiology, implicating anti-angiogenic factors, such as a cleavage product of prolactin, vascular endothelial growth factor (VEGF) inhibitors, and sFLT1, in the pathogenesis of PPCM [4,5].

PPCM typically presents in a similar way to other forms of systolic heart failure. Patients may report symptoms of dyspnea, orthopnea, edema, chest pain, palpitations, and activity intolerance. Unfortunately, many of these symptoms are present in normal pregnancy, which could delay the diagnosis of PPCM. Treatment for PPCM is similar to treatment for other causes of heart failure with reduced ejection fraction (HFrEF). Inotropic agents may be necessary for acute heart failure, and some patients will even require mechanical circulatory support [6]. Outcomes for the majority of patients who develop PPCM are favorable; however, thromboembolic events, fatal arrhythmias, cardiogenic shock, and persistent heart failure do occur [7]. Left ventricular ejection fraction < 30% and left ventricular end-diastolic diameter > 6 cm at the time of presentation have been shown to be predictive of worse prognosis [8].

Currently, there is limited knowledge of the effect of comorbidities on the prognosis of patients with PPCM. Coronary artery disease (CAD) is known to be the most common cause of ischemic cardiomyopathy, causing cell death, fibrosis, and left ventricular dilation [9]. In a study of patients with obstructive CAD undergoing cardiac magnetic resonance imaging for the assessment of left ventricular function, 8.1% were found to have non-ischemic cardiomyopathy, which was associated with a worse overall prognosis compared to ischemic cardiomyopathy [10]. Moreover, CAD has been associated with increased all-cause mortality in patients with all forms of HFrEF, with heart failure exacerbation being the most common cause of death [11]. In patients specifically with non-ischemic cardiomyopathy, coronary atherosclerotic burden is associated with increased major adverse cardiac events, including myocardial infarction [12]. In this study, we aim to evaluate the association of CAD with comorbidities and outcomes in patients with PPCM.

## Materials and methods

Data acquisition

This is a retrospective database study of the National Inpatient Sample (NIS) database. The NIS is part of the Healthcare Cost and Utilization Project (HCUP) set forth by the Agency for Healthcare Research and Quality. It utilizes the International Classification of Diseases, Tenth Edition, Clinical Modification (ICD-10-CM) codes for diagnosis and procedures. The dataset was utilized to examine patients admitted between the years 2016 and 2019. Encounters with primary diagnosis of PPCM were selected using ICD-10 code O90.3. This cohort of patients was further divided into patients with CAD versus patients without CAD. Adult patients ≥ 18 years old were included. We abstracted data from 4769 charts, excluded 39, and were left with 4730 charts for analysis. Institutional Review Board approval was not required as the NIS database provides de-identified patient information.

Outcomes and variables

Patient baseline characteristics such as age, race, and insurance status were extracted. Comorbidities, hospital complications, mortality rates, disposition status, length of stay, and total charges were also analyzed.

The primary aim of the study was to assess whether or not there is a difference in outcomes (mortality, in-hospital complications, length of stay, total charges) between the cohort of patients with PPCM and CAD vs. patients with PPCM and without CAD. We also analyzed the independent association of CAD with outcomes taking into account confounders such as age, race, and comorbidities.

Statistical analysis

Categorical values were analyzed via Pearson chi-square analysis and continuous variables were analyzed via independent Student’s t-test. Logistic regression was performed to generate an odds ratio with 95% confidence intervals (CIs) to assess predictors of mortality in patients with PPCM. We also used logistic regression to assess the independent association of CAD with clinical outcomes after controlling for confounders like age, race, and comorbidities. A p-value of <0.05 was considered statistically significant. All analyses were completed using IBM SPSS Statistics for Windows, version 29.0 (IBM Corp., Armonk, NY).

## Results

There was a total of 4,730 patients with PPCM who met inclusion criteria, 146 of whom had CAD (3.1%). A statistical analysis of baseline characteristics is summarized in Table 1. Patients with CAD were found to be significantly older than their counterparts (37.74 ± 8.42 years vs. 31.89 ± 7.09 years, p = 0.001). Patient disposition (p = 0.001), primary expected payer (p = 0.023), and race (p = 0.043) were all associated with CAD status. Patients with CAD were more likely to be discharged with home health care compared to patients without CAD (32 (21.9%) vs. 386 (8.4%), p = 0.001). The black race was the most frequent in both groups. A total of 81 (57%) CAD patients identified as black compared to 1953 (44%) patients without CAD. More patients with CAD used Medicare as primary payment compared to patients without CAD (25 (17.1%) vs. 402 (8.8%), p = 0.023).

**Table 1:** Baseline characteristics of the study population of PPCM patients stratified according to with and without CAD. Data are presented as n (%) unless otherwise indicated. PPCM: peripartum cardiomyopathy; CAD: coronary artery disease.

Variable	No CAD, n (%)	CAD, n (%)	P-value
Age at admission	31.89 ± 7.09	37.74 ± 8.42	0.001
Race	-	-	0.043
White	1683 (37.9%)	40 (28.2%)	
Black	1953 (44.0%)	81 (57.0%)	
Hispanic	485 (10.9%)	14 (9.9%)	
Asian or Pacific Islander	125 (2.8%)	1 (0.7%)	
Native American	53 (1.2%)	1 (0.7%)	
Other	142 (3.2%)	5 (3.5%)	
Primary expected payer	-	-	0.023
Medicare	402 (8.8%)	25 (17.1%)	
Medicaid	2412 (52.7%)	73 (50.0%)	
Private insurance	1507 (32.9%)	41 (28.1%)	
Self-pay	140 (3.1%)	5 (3.4%)	
No charge	7 (0.2%)	0 (0.0%)	
Other	109 (2.4%)	2 (1.4%)	
Disposition of patient	-	-	0.001
Routine	3731 (81.4%)	102 (69.9%)	
Transfer to a short-term hospital	211 (4.6%)	8 (5.5%)	
Transfer, other	89 (1.9%)	3 (2.1%)	
Home health care	386 (8.4%)	32 (21.9%)	
Against medical advice	100 (2.2%)	0 (0.0%)	
Died in hospital	66 (1.4%)	1 (0.7%)	

Univariate analysis results showing the associations between several comorbidities and CAD in PPCM are depicted in Table 2. Patients with CAD were more likely to have the following conditions as compared to patients without CAD: coagulopathy (9 (6.2%) vs. 142 (3.1%), p = 0.038), type 2 diabetes mellitus (40 (27.4%) vs. 422 (9.2%), p = 0.001), hypertension (27 (18.5%) vs. 318 (6.9%), p = 0.001), peripheral vascular disease (2 (1.4%) vs. 2 (<1%), p = 0.001), atrial fibrillation (23 (15.8%) vs. 235 (5.1%), p = 0.001), pulmonary hypertension (31 (21.2%) vs. 511 (11.1%), p = 0.001), tobacco use disorder (4 (2.7%) vs. 29 (0.6%), p = 0.003), obstructive sleep apnea (17 (11.6%) vs. 232 (5.1%), p = 0.001), chronic kidney disease (32 (21.9%) vs. 347 (7.6%), p = 0.001), and cocaine use disorder (5 (3.4%) vs. 54 (1.2%), p = 0.016). Patients with CAD were less likely to have liver disease as compared to those without CAD (0 (0%) vs. 179 (3.9%), p = 0.015).

**Table 2:** Prevalence of comorbidities in the study population of PPCM patients with and without CAD. PPCM: peripartum cardiomyopathy; CAD: coronary artery disease; IDA: iron deficiency anemia; T2DM: type 2 diabetes mellitus; HTN: hypertension; PVD: peripheral vascular disease; AF: atrial fibrillation; TUD: tobacco use disorder; OSA: obstructive sleep apnea; CUD: cocaine use disorder; OUD: opioid use disorder; CKD: chronic kidney disease; COPD: chronic obstructive pulmonary disease; AUD: alcohol use disorder (AUD); PVD: peripheral vascular disease.

Variable	No CAD, n(%)	CAD, n(%)	P-value
COPD	767 (16.7%)	32 (21.9%)	0.1
Coagulopathy	142 (3.1%)	9 (6.2%)	0.038
T2DM	422 (9.2%)	40 (27.4%)	0.001
HTN	318 (6.9%)	27 (18.5%)	0.001
AUD	40 (0.9%)	3 (2.1%)	0.138
Liver disease	179 (3.9%)	0 (0.0%)	0.015
PVD	2 (0.0%)	2 (1.4%)	0.001
AF	235 (5.1%)	23 (15.8%)	0.001
Hypothyroidism	243 (5.3%)	10 (6.8%)	0.413
Pulmonary hypertension	511 (11.1%)	31 (21.2%)	0.001
TUD	29 (0.6%)	4 (2.7%)	0.003
OSA	232 (5.1%)	17 (11.6%)	0.001
IDA	587 (12.8%)	16 (1%)	0.51
CKD	347 (7.6%)	32 (21.9%)	0.001
Gestational diabetes	135 (2.9%)	3 (2.1%)	0.529
Gestational hypertension	200 (4.4%)	2 (0.4%)	0.078
CUD	54 (1.2%)	5 (3.4%)	0.016
OUD	100 (2.2%)	4 (2.7%)	0.651
Obesity	499 (10.9%)	21 (14.4%)	0.183

A summary of crude analysis of outcomes of PPCM patients with and without CAD is included in Table 3. Patients with CAD were more likely to experience cardiogenic shock (18 (12.3%) vs. 259 (5.7%), p = 0.001), ventricular fibrillation (12 (8.2%) vs. 83 (1.8%), p = 0.001), ventricular tachycardia (22 (15.1%) vs. 287 (6.3%), p = 0.001), cardiac arrest (8 (5.5%) vs. 80 (1.7%), p = 0.001), ST-segment elevation myocardial infarction (STEMI) (9 (6.2%) vs. 1 (<1%), p = 0.001), and non-ST elevation myocardial infarction (NSTEMI) (17 (11.6%) vs. 145 (3.2%), p = 0.001) as compared to patients without CAD. Patients with CAD were also more likely to have left heart (36 (24.7%) vs. 105 (2.3%), p = 0.001) and right heart (14 (9.6%) vs. 224 (4.9%), p = 0.011) catheterizations. Patients without CAD were more likely to have preeclampsia than those with CAD (4 (2.7%) vs. 676 (14.7%), p = 0.001). Other obstetric complications such as eclampsia, post-partum hemorrhage, and HELLP (hemolysis, elevated liver enzymes, and low platelets) syndrome were independent of CAD status.

**Table 3:** Outcomes of the study population of PPCM patients with and without CAD. Data are presented as n (%). PPCM: peripartum cardiomyopathy; CAD: coronary artery disease; PPM: permanent pacemaker; VF: ventricular fibrillation; VT: ventricular tachycardia; RHC: right heart catheterization; LHC: left heart catheterization; IABP: intra-aortic balloon pump; STEMI: ST-segment elevation myocardial infarction; NSTEMI: non-ST elevation myocardial infarction; HELLP: hemolysis, elevated liver enzymes, and low platelets.

Variable	No CAD, n (%)	CAD, n (%)	P-value
Died during hospitalization	66 (1.4%)	1 (0.7%)	0.447
Length of stay (days)	6	7	0.339
Total charges ($)	87,413	105,948	0.391
PPM	5 (0.1%)	0 (0.0%)	0.69
Cardiogenic shock	259 (5.7%)	18 (12.3%)	0.001
Vasopressor	49 (1.1%)	3 (2.1%)	0.261
IABP	82 (1.8%)	5 (3.4%)	0.148
Mechanical ventilation	151 (3.3%)	2 (1.4%)	0.196
VF	83 (1.8%)	12 (8.2%)	0.001
VT	287 (6.3%)	22 (15.1%)	0.001
LHC	105 (2.3%)	36 (24.7%)	0.001
RHC	224 (4.9%)	14 (9.6%)	0.011
Preeclampsia	676 (14.7%)	4 (2.7%)	0.001
Cardiac arrest	80 (1.7%)	8 (5.5%)	0.001
Eclampsia	70 (1.5%)	2 (1.4%)	0.67
HELLP	41 (0.9%)	0 (0,0%)	0.251
Shock after delivery	68 (1.5%)	2 (1.4%)	0.911
Post-partum hemorrhage	163 (3.6%)	1 (0.7%)	0.062
STEMI	1 (0.0%)	9 (6.2%)	0.001
NSTEMI	145 (3.2%)	17 (11.6%)	0.001

Multivariable logistic regression was used to evaluate the independent association of CAD with in-hospital outcomes after controlling for confounders such as age, race, and comorbidities. STEMI (adjusted odds ratio (aOR): 58.457, 95% CI: 5.403-632.504, p = 0.001) and left heart catheterization (aOR: 10.178, 95% CI: 6.008-17.244, p = 0.001) were positively associated with CAD. Conversely, we found CAD to be protective against preeclampsia (aOR: 0.351, 95% CI: 0.126-0.979, p = 0.045).

**Figure 1:**
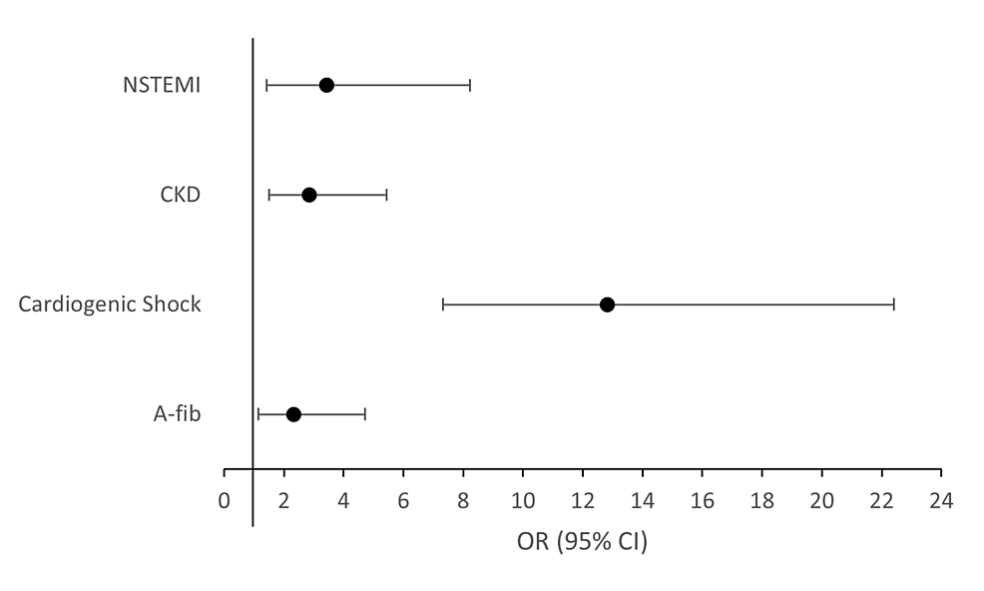
Predictors of mortality in the study population of PPCM patients. PPCM: peripartum cardiomyopathy; CKD: chronic kidney disease; A-fib: atrial fibrillation; NSTEMI: non-ST elevation myocardial infarction.

We conducted a second multivariable logistic regression to evaluate comorbidities and demographic variables as predictors of in-hospital mortality for patients with PPCM, as summarized in Figure 1. Predictors of mortality include cardiogenic shock (aOR: 12.818, 95% CI: 7.332-22.411, p = 0.001), NSTEMI (aOR: 3.429, 95% CI: 1.43-8.22, p = 0.006), chronic kidney disease (aOR: 2.851, 95% CI: 1.495-5.435, p = 0.001), and atrial fibrillation (aOR: 2.326, 95% CI: 1.145-4.723, p = 0.020).

## Discussion

This was a retrospective database study of the relationship of CAD with outcomes in patients with PPCM. PPCM patients with comorbid CAD were on average older than patients without CAD. This was expected given the fact that CAD prevalence increases with age. Conditions such as type 2 diabetes mellitus, hypertension, peripheral vascular disease, tobacco use disorder, obstructive sleep apnea, and cocaine use disorder that are often comorbid with CAD in the general population were also comorbid with CAD in patients with PPCM [13-15].

Although the presence of CAD was not independently predictive of mortality in our study, it was positively associated with acute coronary syndrome, cardiac arrest, and cardiogenic shock during hospitalization. Studies have implicated the role of coronary atherosclerosis with non-ischemic cardiomyopathy [10,16]. In non-ischemic cardiomyopathy, comorbid CAD has been shown to be associated with an increased incidence of major adverse cardiovascular events, including acute heart failure and acute coronary syndrome [12]. Not surprisingly, acute coronary syndrome and cardiogenic shock were predictive of in-hospital mortality for patients with PPCM. Chronic kidney disease (CKD) and atrial fibrillation were also predictive of mortality for patients with PPCM. CKD and heart failure are known to have synergistic effects due to the activation of the renin-angiotensin-aldosterone system, fluid overload, and sympathetic activation, which worsen the severity of both diseases [17]. Studies have shown atrial fibrillation to also be associated with increased mortality in patients with heart failure, regardless of whether it develops before or after heart failure [18]. In addition, the association between atrial fibrillation and outcomes in PPCM has been studied before and showed that atrial fibrillation was associated with increased mortality [19].

Preeclampsia was the only obstetric complication that was associated with CAD in PPCM. Patients with CAD were less likely to have preeclampsia than those without CAD. It is well documented that preeclampsia is a risk factor for developing PPCM, with the production of anti-angiogenic factors being a commonality in the possible pathogenesis of these conditions [20]. There is no clear mechanistic explanation for why the presence of CAD was associated with a reduced likelihood of preeclampsia. However, it is known that patients with CAD develop collateral circulation in response to ischemia via activation of angiogenic pathways [21]. It is possible that activation of these pro-angiogenic factors in CAD has a protective effect against developing preeclampsia. This mechanism may also be the reason why CAD was not associated with increased mortality in PPCM, despite its association with worse outcomes in other forms of systolic heart failure [11].

Limitations of our study include the fact that this is an observational study; therefore, we can only infer correlations and not causations. Patients were not followed longitudinally or in the outpatient settings; therefore, long-term outcomes cannot be established. The sample size for the cohort of patients with CAD was small compared to their counterparts, which may lead to higher variability. In addition, existing confounders such as medication use are not provided by the NIS database and were not accounted for in our study. Lastly, the NIS database does not make it feasible to match patients with PPCM based on their trimester of pregnancy.

## Conclusions

In this study, we found that in women with PPCM, CAD was independently associated with STEMI and left heart catheterization. In addition, we found predictors of mortality in PPCM to include chronic kidney disease, atrial fibrillation, NSTEMI, and cardiogenic shock. Furthermore, our results show that CAD was protective against preeclampsia. There may be an interesting biochemical mechanism based on the activation of pro-angiogenic pathways in CAD that lowers the likelihood of developing preeclampsia. This relationship should be explored further, as it may provide insight into the pathophysiology of PPCM.
